# Assessing the role of skin complexion in determining tooth shade, and gingival pigmentation in dental aesthetics

**DOI:** 10.12669/pjms.41.5.11824

**Published:** 2025-05

**Authors:** Rizwan Jouhar

**Affiliations:** 1 Rizwan Jouhar Department of Restorative Dental Sciences, College of Dentistry, King Faisal University, 31982 Al-Ahsa, Saudi Arabia

**Keywords:** Gingival pigmentation, Skin complexion, Tooth shade, Dental Aesthetics

## Abstract

**Background & Objective:**

Skin complexion and oral aesthetics significantly influence facial harmony and smile attractiveness. This study aimed to explore the relationship between skin tone, gingival pigmentation, and tooth shade to achieve natural and aesthetically pleasing dental outcomes.

**Methods:**

This cross-sectional observational study was conducted at the Dental Clinic Complex of King Faisal University, Al-Ahsa from September 2024 to January 2025. A total of 261 participants of both genders, with good oral health and healthy central incisors for shade selection, were included. Skin complexion was assessed using the Revlon (Revlon Foundation Makeup Shade Guide, USA), while gingival pigmentation with the Dummett-Gupta Oral Pigmentation Index, and tooth shade using the Vita Easyshade^®^ V (Bad Säckingen, Germany). Data analysis was performed using SPSS (Version 20.0, SPSS Inc., Chicago), with chi-square, Spearman’s correlation, and linear regression tests. A p-value ≤0.05 was considered significant.

**Results:**

Most participants were male (65.5%). Shade A was the most common tooth shade (62.1%), followed by shade B (30.3%). Medium skin tone (51.0%) was predominant, with dark (28.0%) and fair (21.0%) tones following. The majority had no gingival pigmentation (66.7%). A significant association was found between skin complexion and tooth shade (p = 0.018), with a weak positive correlation (ρ = 0.146, p = 0.019). No significant associations were observed with gingival pigmentation or smile satisfaction.

**Conclusions:**

Fair-skinned participants tended to have lighter tooth shades, whereas those with medium and dark skin tones had relatively darker shades. However, gingival pigmentation and smile satisfaction showed no significant correlation with skin complexion.

## INTRODUCTION

Gingival tissues, in harmony with the face, lips, and teeth, are integral to dentofacial aesthetics. Modern dentistry emphasizes achieving a balanced dental appearance that incorporates both teeth and gingiva to enhance smile aesthetics. Designing an aesthetically pleasing smile remains a significant challenge for dentists, as it involves aligning tooth color with various facial features such as hair, complexion, eyes, and age.^1^ Teeth and skin tone significantly influence facial aesthetics, impacting social interactions and self-confidence. Tooth color, a key element of dental appeal, is shaped by factors such as age, diet, and genetics. Selecting the right tooth shade is critical in dental treatments, often relying on shade guides to ensure natural dental aesthetics.^2^

Skin color also impacts the perceived attractiveness of teeth. Studies suggest a correlation between tooth shade and skin tone, with lighter skin tones often linked to lighter tooth shades.^3^ Understanding this relationship is crucial for selecting prosthetic teeth or restorative materials to achieve a natural appearance that harmonizes with the patient’s facial complexion.^4^ Furthermore, tooth color perception is subjective, influenced by surrounding facial features and cultural preferences.^5^ Aging affects both teeth and skin, with teeth darkening due to enamel thinning and external staining, while skin undergoes texture, pigmentation, and elasticity changes.^4^ The interplay between teeth and skin appearance is thus central to achieving optimal facial aesthetics.

Previous studies on smile attractiveness have focused on populations from the Italy, and Europe.^2^,^6^ Although some research has focused on South Indian populations,^7^,^8^ the influence of skin tone and tooth shade on the smile aesthetics of native South Indians remains underexplored. Additionally, few studies have comprehensively investigated the relationship between tooth color and skin tone, which is crucial for selecting prosthetic teeth.^9^ Oral hyperpigmentation, an excess of pigmentation in the oral cavity, primarily affects gingival tissues and is more prevalent in dark-skinned populations.^10^,^11^ It is often hereditary and termed physiological pigmentation, caused by increased melanin production.^12^ Facial skin tone correlates strongly with gingival pigmentation, with fair skin typically showing mild pigmentation, while darker skin tones experience moderate to severe pigmentation.^6^,^13^ The correlation between facial skin tone and gingival pigmentation suggests the importance of considering these factors in aesthetic dentistry.^14^

In Saudi Arabia, the diverse range of skin tones, ethnicities, and social contexts provides a unique backdrop for studying the interaction between facial skin complexion, gingival pigmentation, and tooth shade. Facial aesthetics, including the harmony between tooth shade, gingival color, and skin complexion, are critical to self-esteem and social interactions. Despite its importance, limited research has examined the interaction between facial complexion and oral aesthetics, particularly in achieving a harmonious balance between gingival and tooth shades. This study aims to address this gap by investigating the relationships between skin complexion, gingival pigmentation, and tooth shade within a Saudi Arabian cohort. The null hypothesis is that there is a significant association between skin complexion and tooth shade selection. The findings aimed to inform culturally and regionally specific aesthetic dentistry practices that align with the needs and preferences of this population.

## METHODS

This cross-sectional observational study was conducted at the Dental Clinic Complex of King Faisal University, Al-Ahsa, from September 2024 to January 2025 using a convenience sampling technique. The sample size for this study was determined with Open EPI software using a 95% confidence level, a 5% margin of error, and 80% power of the test. Considering the gingival pigmentation rate (25.4%)^14^ occurrence in the attached gingiva and interdental papilla. A minimum of 261 participants were included in the study. A total of 261 male and female participants, aged 18 to 70 years, were included. Eligibility criteria required participants to have good oral health and at least one central incisor free from restorations, caries, or stains for accurate shade measurement. Individuals with skin conditions affecting facial complexion, poor oral health, ongoing orthodontic treatment, visible dental restorations or prostheses, or other conditions impacting complexion were excluded.

### Ethical Approval:

This study was approved by the institutional Ethics Committee Ref., (KFU-REC-2024-SEP-ETHICS2527, September 18, 2024).

### Data Collection Procedure:

Informed consent was obtained from all participants prior to the study. The Vita Easyshade® V (Bad Säckingen, Germany) device was used to determine the shade of the upper right central incisor. To ensure accuracy, shade readings were conducted at the beginning of appointments to avoid the effects of dehydration. Facial skin complexion was assessed using the Ideal Balance Quick Stick Makeup Shade by Revlon (Revlon Foundation Makeup Shade Guide, USA). Skin tones were categorized as Fair, Medium, and Dark. The fair skin group included Revlon shades Vanilla, Shell, and Nude, while the medium skin group comprised Natural Beige, Medium Beige, and Cool Beige. The dark skin group included Golden Beige and Rich Ginger shades. Skin tone assessments were performed on the back of participants’ hands, ensuring the areas were free of makeup or residue.

**Fig.1:**
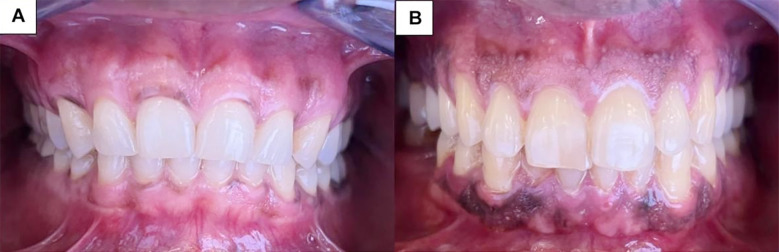
“A” represents Class-VI (pigmentation in both the marginal gingiva and interdental papilla) and “B” represents Class-II (pigmentation in the attached gingiva and interdental papilla)

Gingival tissue pigmentation was evaluated by examining the location, and extent. It includes six classes: Class-I (pigmentation confined to the attached gingiva), Class-II (pigmentation in the attached gingiva and interdental papilla), Class-III (diffuse pigmentation involving all parts of the gingiva), Class-IV (pigmentation in the marginal gingiva only), Class-V (pigmentation in the interdental papilla only), and Class-VI (pigmentation in both the marginal gingiva and interdental papilla)^15^ (Fig.1). The intensity of physiological gingival pigmentation was measured using the Dummett-Gupta Oral Pigmentation Index (DOPI) as mentioned in Table-I.^16^

**Table I:** Dummett-Gupta Oral Pigmentation Index

1.	Pink tissue (No pigmentation)
2.	Light brown tissue (Mild pigmentation).
3.	Medium brown or mixed pink and brown tissue (Moderate pigmentation).
4.	Dark brown or blue/black tissue (Heavy pigmentation).

Smile attractiveness was self-assessed by participants through the question: “How satisfied are you with your smile?” Responses were recorded on a five point scale, where one indicated “very dissatisfied” and five indicated “very satisfied.”

### Statistical analysis:

The data was analyzed using the Statistical Package for Social Sciences (SPSS) for Windows (Version 20.0, SPSS Inc., Chicago). Demographic information, including skin complexion, gingival pigmentation, and tooth shade distributions, was summarized as frequencies and percentages. Shapiro-Wilk test was used to analyze data distribution. The chi-square test was employed to assess associations between gingival pigmentation, tooth shade, and skin complexion. Spearman’s rank correlation coefficient was used to evaluate the relationships among skin complexion, gingival pigmentation, and tooth shade. Simple linear regression analysis was conducted to determine the effect of skin complexion on perceived smile attractiveness. A p-value of <0.05 was considered statistically significant

## RESULTS

The study included 261 participants with a nearly even distribution across age groups: 123 (47.1%) were aged 18–30 years, 78 (29.9%) were aged 31–50 years, and 60 (23.0%) were aged 51 years and older. Male participants comprised the majority, 171 (65.5%), while females accounted for 90 (34.5%). Most participants were Saudi nationals (194, 74.3%), with non-Saudis making up 67 (25.7%).

### Tooth shade distribution:

**Fig.2:**
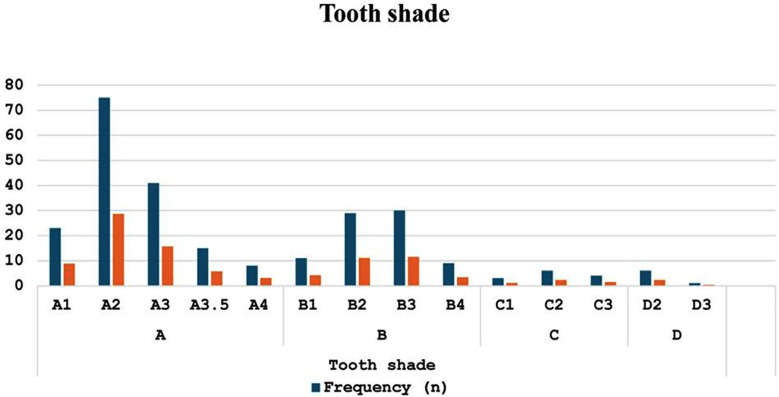
The distribution of tooth shade among the participants.

Tooth shade analysis revealed that shade A was the most common, seen in 162 (62.1%) participants, followed by shade B in 79 (30.3%). Shades C and D were less frequent, observed by 13 (5.0%) and 7 (2.7%) participants, respectively (Table-II). Among specific shades, A2 was the most prevalent (75, 28.7%), followed by A3 (41, 15.7%). Other shades in the A category included A1 (23, 8.8%), A3.5 (15, 5.7%), and A4 (8, 3.1%). Shades in the B category included B3 (30, 11.5%) and B2 (29, 11.1%), while C and D shades were relatively rare (Fig.2).

**Table-II:** Demographic details of Participants (n=261).

Variables		Frequency (n)	Percentage (%)
Age group (years)	18-30	123	47.1
	31-50	78	29.9
	51 and above	60	23.0
Gender	Male	171	65.5
	Female	90	34.5
Nationality	Saudi	194	74.3
	Non-Saudi	67	25.7
Tooth shade	A	162	62.1
	B	79	30.3
	C	13	5.0
	D	7	2.7

### Skin complexion and Gingival pigmentation:

**Fig.3:**
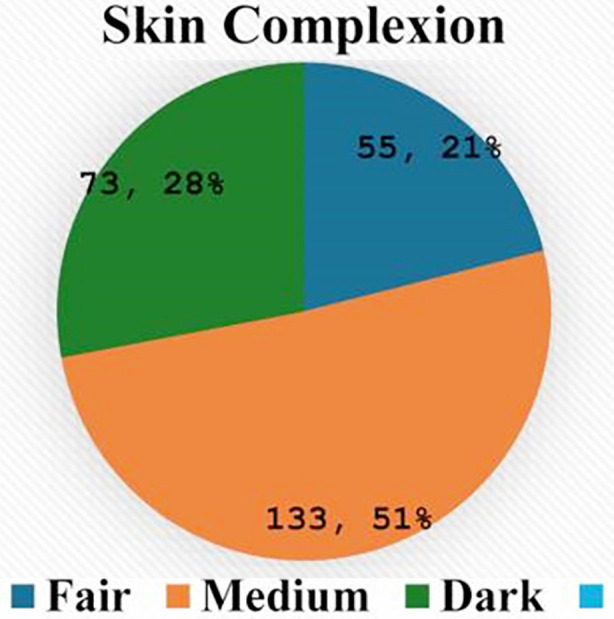
The distribution of skin complexion among the participants.

Skin complexion analysis showed that 133 (51.0%) participants had medium skin tones, followed by dark skin (73, 28.0%) and fair skin (55, 21.0%) (Fig.3). The Dummett-Gupta Oral Pigmentation Index (DOPI) indicated that 174 (66.7%) participants had no gingival pigmentation, 51 (19.5%) had mild pigmentation, 25 (9.6%) had moderate pigmentation, and 11 (4.2%) had severe pigmentation. Gingival pigmentation was distributed as follows: Class-I (11, 4.2%), Class-II (22, 8.4%), Class-III (12, 4.6%), Class-IV (18, 6.9%), Class-V (14, 5.4%), and Class-VI (10, 3.8%).

Participants with fair skin showed the highest proportion of no pigmentation (41, 74.5%), followed by those with medium (93, 69.9%) and dark skin (40, 54.8%). Mild pigmentation was more common in participants with dark skin (17, 23.3%) compared to medium (25, 18.8%) and fair (9, 16.4%) complexions. Moderate pigmentation was most common in dark skin participants (10, 13.7%). Severe pigmentation was absent in fair skin participants but present in medium (5, 3.8%) and dark skin (6, 8.2%) groups. These differences were not statistically significant (p = 0.229).

### Skin complexion and Tooth shade:

Tooth shade distribution showed a significant association with skin complexion (p = 0.018). Participants with fair skin predominantly had shade A (45, 81.8%), compared to medium (76, 57.1%) and dark skin (41, 56.2%). Shades in the B category were more frequent in participants with dark (29, 39.7%) and medium skin (43, 32.3%) compared to fair skin (7, 12.7%). Shades C and D were rare across all groups (Table-III).

**Table-III:** The association of skin complexion with tooth shade and gingival pigmentation.

Variables		Skin complexion			
		Fair	Medium	Dark	p-value
Dummett-Gupta Oral Pigmentation Index (DOPI)	No Pigmentation	41(74.5%)	93(69.9%)	40(54.8%)	0.229
	Mild Pigmentation	9(16.4%)	25(18.8%)	17(23.3%)	
	Moderate Pigmentation	5(9.1%)	10(7.5%)	10(13.7%)	
	Severe Pigmentation	0(0.0%)	5(3.8%)	6(8.2%)	
Tooth shade by Vita shade guide	A	45(81.8%)	76(57.1%)	41(56.2%)	0.018
	B	7(12.7%)	43(32.3%)	29(39.7%)	
	C	2(3.6%)	9(6.8%)	2(2.7%)	
	D	1(1.8%)	5(3.8%)	1(1.4%)	

### Smile satisfaction:

Most participants (182, 69.7%) were satisfied with their smiles, while 52 (19.9%) reported dissatisfaction, and 15 (5.7%) were very dissatisfied. A small proportion rated their satisfaction as neutral (6, 2.3%) or very satisfied (6, 2.3%) (Table-IV).

**Table-IV:** Distribution of Oral Pigmentation, Gingival Pigmentation Location, and Smile Attractiveness Self-Assessment.

Variables		Frequency (n)	Percentage (%)
Dummett-Gupta Oral Pigmentation Index [DOPI]	No Pigmentation	174	66.7
	Mild Pigmentation	51	19.5
	Moderate Pigmentation	25	9.6
	Severe Pigmentation	11	4.2
Location of gingiva	No pigmentation	174	66.7
	Class-I (Pigmentation on Attached Gingiva Only)	11	4.2
	Class-II (Pigmentation on Attached Gingiva and Interdental Papilla)	22	8.4
	Class-III (Diffuse pigmentation involving all part of gingiva)	12	4.6
	Class-IV (Pigmentation on Marginal Gingiva only)	18	6.9
	Class-V (Pigmentation on Interdental Papilla only)	14	5.4
	Class-VI (Pigmentation on Marginal gingiva and Interdental Papilla)	10	3.8
Self-assessment of smile attractiveness	Very dissatisfied	15	5.7
	Dissatisfied	52	19.9
	Neutral	6	2.3
	Satisfied	182	69.7
	Very satisfied	6	2.3

### Correlations and Predictive analysis:

A weak but statistically significant positive correlation was found between skin complexion and tooth shade (ρ = 0.146, p = 0.019), suggesting that skin complexion influences tooth shade. No significant correlation was observed between skin complexion and gingival pigmentation (ρ = -0.063, p = 0.311) (Table-V). The relationship between skin complexion and smile satisfaction showed no statistical significance (R² = 0.001, F = 0.343, p = 0.559), indicating that skin complexion is not a meaningful predictor of smile satisfaction in this population (Table-VI). This may suggest that other factors, such as tooth alignment and overall facial harmony, play a larger role in perceived smile attractiveness.

**Table-V:** Correlation of skin complexion with tooth shade and gingival pigmentation.

Variable	Skin Complexion	
	ρ	p-value
Tooth shade	0.146	0.019
Gingival pigmentation	- 0.063	0.311

Spearman’s rank correlation coefficient.

**Table-VI:** Impact of skin complexion on the perceived attractiveness of smiles.

Variables	Smile satisfaction						
	R Square	F-Statistic	p-value (F-Test)	Unstandardized Coefficient (B)	Standard Error (B)	95% Confidence Interval for B	p-value
Skin complexion	0.001	0.343	0.559	-0.053	0.091	[-0.231, 0.125]	0.559

Simple linear Regression.

## DISCUSSION

This study found a significant association between skin complexion and tooth shade (p = 0.018), with fair-skinned individuals predominantly exhibiting lighter tooth shades, while medium and dark skin tones were linked to darker shades. Hence, the null hypothesis accepted in this study. However, no significant correlation was observed between skin complexion and gingival pigmentation or smile satisfaction. Most participants (69.7%) were satisfied with their smiles, regardless of skin tone. A weak but statistically significant positive correlation was identified between skin complexion and tooth shade (ρ = 0.146, p = 0.019), indicating that skin tone may influence tooth color selection in aesthetic dentistry.

Selecting an appropriate tooth shade is a meticulous process requiring an understanding of color parameters such as hue, chroma, value, and translucency. Adherence to standardized procedures is essential to achieve a natural appearance that complements facial features and aligns with the patient’s skin tone.^17^ This study examined the relationship between tooth shade, gingival pigmentation, and skin complexion, aiming to inform aesthetic dental practices.

The selection of tooth shade significantly impacts patients’ aesthetic satisfaction. In this study, medium skin tone was the most prevalent (51.0%), followed by dark (28.0%) and fair complexions (21.0%). Shade A was the most common tooth shade (62.1%), with A2 (28.7%) being particularly dominant. Gingival pigmentation was absent in most participants (66.7%), with mild to severe pigmentation more common in individuals with darker skin tones.

However, these findings differ from a previous cross-sectional study where lighter tooth shades (e.g., B1) were prevalent across all skin tones.^18^ This contrast highlights the variability in tooth shade distribution between populations. Skin color often correlates with gingival pigmentation intensity. A study involving 391 participants reported a statistically significant relationship between skin color and gingival pigmentation, with medium brown skin and tooth shade A1 being most common among those with pigmentation.^19^ Similarly, Ponnaiyan et al.^15^ found a significant correlation between skin color and gingival pigmentation in South Indians. However, in the present study, gingival pigmentation varied across skin tones but was not statistically significant (p = 0.229). Participants with fair skin predominantly had no pigmentation (74.5%), while severe pigmentation was more frequent in those with dark skin (8.2%).

In Saudi populations, gender and skin tone influence tooth shade preferences, with lighter-skinned individuals preferring lighter tooth shades.^20^ While previous studies suggested that people with medium-to-dark skin tones often have lighter teeth, the current study found a significant association between skin complexion and tooth shade (p = 0.018). Participants with fair skin predominantly exhibited tooth shades from the A group, which represents reddish-brown hues, while those with darker skin tones were more likely to exhibit shades from the B group, representing reddish-yellow hues. These findings emphasize the importance of considering skin tone when selecting tooth shades for aesthetic procedures.

This study observed no significant association between skin complexion and gingival pigmentation or smile satisfaction. Most participants expressed satisfaction with their smiles, regardless of skin tone. This contrasts with other studies suggesting that smile attractiveness is influenced by both tooth shade and surrounding skin color.^21^ However, the preference for lighter tooth shades in this study aligns with previous research indicating a universal inclination toward lighter teeth across populations.^20^-^23^

A weak but statistically significant positive correlation was identified between skin complexion and tooth shade (ρ = 0.146, p = 0.019). This aligns with previous findings highlighting a relationship between tooth shade and skin color. For example, research in the United States found that lighter skin tones were associated with darker teeth, while Indian studies reported that lighter skin tones correlated with lighter tooth shades.^22^,^24^ Similarly, a study in a Jordanian population identified significant differences in tooth shade across skin color groups, with darker-skinned individuals more likely to have lighter teeth.^25^ In contrast, the present study found a weak positive correlation, indicating that fairer skin tones were associated with lighter tooth shades.

### Limitations:

Due to its cross-sectional design, this study cannot establish causality. Additionally, the predominantly male, Saudi sample may limit the generalizability of the findings. Self-reported smile satisfaction introduces potential bias. Future research should adopt more diverse samples, longitudinal designs to explore causal relationships, and objective measures of smile satisfaction. Investigating additional factors, such as age and oral health, may also provide further insights into tooth shade selection and aesthetic outcomes.

## CONCLUSION

This study found that fair-skinned participants predominantly exhibited lighter tooth shades (A category), while medium and dark skin tones were associated with darker shades (B category). These findings indicate that while skin complexion affects tooth shade selection, it does not impact gingival pigmentation or smile satisfaction.

## References

[1] Ho DK, Ghinea R, Herrera LJ, Angelov N, Paravina RD (2015). Color Range and Color Distribution of Healthy Human Gingiva:a Prospective Clinical Study. Sci Rep.

[2] Van der Geld P, Oosterveld P, Van Heck G, Kuijpers-Jagtman AM (2007). Smile attractiveness. Self-perception and influence on personality. Angle Orthod.

[3] Samorodnitzky-Naveh GR, Geiger SB, Levin L (2007). Patients'satisfaction with dental esthetics. J Am Dent Assoc.

[4] Haralur SB, Dibas AM, Almelhi NA, Al-Qahtani DA (2014). The Tooth and Skin Colour Interrelationship across the Different Ethnic Groups. Int J Dent.

[5] Ahmed MA (2024). Assessing the shade matching accuracy among dental students through visual and instrumental methods. Pak J Med Sci.

[6] Horn S, Matuszewska N, Gkantidis N, Verna C, Kanavakis G (2021). Smile dimensions affect self-perceived smile attractiveness. Sci Rep.

[7] Veeraganta SK, Savadi RC, Baroudi K, Nassani MZ (2015). Differences in tooth shade value according to age, gender and skin color:A pilot study. J Indian Prosthodont Soc.

[8] Sharma V, Punia V, Khandelwal M, Punia S, Lakshmanarao B (2010). A study of relationship between skin color and tooth shade value in population of Udaipur, Rajasthan. Int J Dent Clin.

[9] Pustina-Krasniqi T, Xhajanka E, Ajeti N, Bicaj T, Dula L, Lila Z (2018). The relationship between tooth color, skin and eye color. Eur Oral Res.

[10] Shenawy H, Fahd A, Ellabban M, Dahaba M, Khalifa M (2017). Lasers for Esthetic Removal of Gingival Hyperpigmentation:A Systematic Review of Randomized Clinical Trials. Int J Adv Res.

[11] Mahmood AS, Diab BS (2024). Gingival pigmentation in relation to anti-inflammatory salivary interleukin 10, a comparative study. J Pak Med Assoc.

[12] Farid H, Shinwari MS, Khan FR, Tanwir F (2017). Journey from black to pink gums:management of melanin induced physiological gingival hyper pigmentation. J Ayub Med Coll Abbottabad.

[13] Kim HK (2018). A study on the color distribution of natural teeth by age and gender in the Korean population with an intraoral spectrophotometer. J Esthet Restor Dent.

[14] Ghani B, Jouhar R, Ahmed N (2016). Relationship of facial skin complexion with gingiva and tooth shade on smile attractiveness. JBR J Interdiscip Med Dent Sci.

[15] Ponnaiyan D, Jegadeesan V, Perumal G, Anusha A (2014). Correlating skin color with gingival pigmentation patterns in South Indians –a cross sectional study. Oral Health Dent Manag.

[16] Peeran SW, Ramalingam K, Peeran SA, Altaher OB, Alsaid FM, Mugrabi MH (2014). Gingival pigmentation index proposal of a new index with a brief review of current indices. Eur J Dent.

[17] The Glossary of Prosthodontic Terms:Ninth Edition (2017). J Prosthet Dent.

[18] Pradhan D, Shrestha L, Lohani J (2020). Tooth Shade and Skin Colour:A Descriptive Cross-Sectional Study. J Nepal Med Assoc.

[19] Mirdad A, Alqarni M, Bukhari A, Alaqeely R (2023). Gingival Pigmentation Features in Correlation with Tooth and Skin Shades:A Cross-Sectional Study in a Saudi Population. Oral Health Prev Dent.

[20] Labban N, Al-Otaibi H, Alayed A, Alshankiti K, Al-Enizy MA (2017). Assessment of the influence of gender and skin color on the preference of tooth shade in Saudi population. Saudi Dent J.

[21] Sabherwal RS, Gonzalez J, Naini FB (2009). Assessing the influence of skin color and tooth shade value on perceived smile attractiveness. J Am Dent Assoc.

[22] Vadavadagi SV, Kumari KV, Choudhury GK, Vilekar AM, Das SS, Jena D (2016). Prevalence of Tooth Shade and its Correlation with Skin Colour - A Cross-sectional Study. J Clin Diagn Res.

[23] Di Murro B, Gallusi G, Nardi R, Libonati A, Angotti V, Campanella V (2020). The relationship of tooth shade and skin tone and its influence on the smile attractiveness. J Esthet Restor Dent.

[24] Jahangiri L, Reinhardt SB, Mehra RV, Matheson PB (2002). Relationship between tooth shade value and skin color:an observational study. J Prosthet Dent.

[25] Al-Nsour HF, Al-Zoubi TT, Al-Rimawi AS (2018). Relationship between tooth value and skin color in patients visiting Royal Medical Services clinics of Jordan. Electron Physician.

